# Learning to suppress a location does not depend on knowing which location

**DOI:** 10.3758/s13414-021-02404-z

**Published:** 2022-02-22

**Authors:** Ya Gao, Jan Theeuwes

**Affiliations:** 1 Department of Experimental and Applied Psychology, Vrije Universiteit Amsterdam, Boechorststraat 7, 1081 BT Amsterdam, the Netherlands; 2 Institute Brain and Behavior Amsterdam (iBBA), Amsterdam, the Netherlands; 3 William James Center for Research, ISPA-Instituto Universitario, Lisbon, Portugal

**Keywords:** Attentional capture, Awareness, Visual search, Statistical regularities

## Abstract

The present study investigated whether explicit knowledge and awareness regarding the regularities present in the display affects statistical learning (SL) in visual search. Participants performed the additional singleton paradigm in which a salient distractor was presented much more often in one location than in all other locations. Previous studies have shown that participants learn this regularity as the location that is most likely to contain a distractor becomes suppressed relative to all other locations. In the current study, after each trial, participants had to either indicate the location of the distractor or the location of the target. Those participants that reported the distractor location, were very much aware of the regularity present in the display. However, participants that reported the target location were basically unaware of the regularity regarding the distractor. The results showed no difference between these groups in the amount of suppression of the high-probability location. This indicates that regardless of whether participants had explicit knowledge or not, the suppression was basically the same. We conclude that explicit knowledge and awareness does not contribute to learning to suppress a location. This conclusion is consistent with the notion that statistical learning is automatic, operating without conscious effort or awareness.

## Introduction

In everyday life, we often effortfully direct our attention to locations in our environment that we know are important. For example, when waiting to pick up a friend at the airport, we may direct our attention to the door of the gate in anticipation of his or her arrival. Alternatively, our attention may be captured automatically by salient events in our environment. For example, flashing gate information, auditory messages, and our phone that is ringing may distract us from our top-down goal to keep an eye on the gate for the arrival of our friend. Adequate attentional selection is crucial and entails the prioritization and suppression of incoming information (Broadbent, 1958). One question that has seldom been addressed is whether this prioritization and suppression of information is an active, explicit processes, or whether it is largely driven by implicit, unconscious processes.

In addition to top-down attention and bottom-up capture, recent studies have shown that lingering biases of previous selection episodes (i.e., selection history) play a major role in attentional selection (Awh et al., 2012; Failing & Theeuwes, 2018; Maljkovic & Nakayama, 2000; Theeuwes, 2018, 2019). Such lingering biases may guide attention towards particular objects that are neither part of the top-down set of the observer (i.e., objects that are irrelevant for the task), nor do they stand out from the environment and capture attention in a bottom-up way (Theeuwes, 2018). The question addressed in the current paper is whether being aware of these lingering biases affect attentional selection.

Lingering biases of attention play a crucial role in those circumstances in which statistical regularities are present in the environment. For example, in contextual cueing task, participants need to detect the “T” shape target among a series of “L”-shaped distractors. Unbeknownst to the participants, half of the displays appear repeatedly with the same configuration of the target and distractors, while the others only appear once during the experiment. Participants learn this spatial regularity as they become faster in identifying the target in the repeated displays in comparison with the novel displays (Chun & Jiang, 1998; Chun & Turk-Browne, 2008). Also, previous studies have shown that the spatial probabilities of target can serve as a powerful attentional bias that produced faster detection of targets in high-probability locations or quadrants than of those in low-probability locations or quadrants (Ferrante et al., 2018; Geng & Behrmann, 2005; Jiang et al., 2013). It is argued that through a process called statistical learning, observers are able to extract the distributional properties from sensory input across space and time (Frost et al., 2015).

In a series of recent studies, Theeuwes and colleagues showed that statistical learning of regularities holds not only for targets but also for distractors (Failing et al., 2019; Gao & Theeuwes, 2020; Wang & Theeuwes, 2018a, 2018b, 2018c). Using the classic additional singleton task, it was shown that target selection was faster when the salient distractor was presented more often in one location than in all other locations. In other words, because the distractor singleton was presented at a high-probability location, it captured less attention than when it was presented at a low-probability location. These findings were interpreted as evidence that implicit statistical regularities regarding the distractor result in biased attention away from locations that are likely to contain the distractor (see also Ferrante et al., 2018; Goschy et al., 2014).

In almost all these studies investigating statistical regularities in visual search, participants were asked at the end of the experiment whether they were aware of the regularities in the display. For example, in contextual cueing studies, at the end of the experiment participants are asked to indicate which displays were repeated during the search experiment. The results showed that participants are usually not above chance level in discriminating between old and new configurations (Chun & Jiang, 2003). Similar findings have been reported with the additional singleton tasks investigating statistical regularities regarding the distractor. Also in these tasks, very few participants explicitly mention that the distractor appears more often in one location than in all other locations (Duncan & Theeuwes, 2020; Theeuwes & Failing, 2020; Wang & Theeuwes, 2018a, 2018b, 2018c). Overall, there is some general agreement that there is usually little, if any, awareness of the regularities present in the display (Chun & Jiang, 1998; Chun & Turk-Browne, 2008; Jiang et al., 2013; Wang & Theeuwes, 2018a, 2018b; for different views, see Smyth & Shanks, 2008; Vadillo et al., 2020). On the basis of this type of data, oftentimes it is concluded that through statistical learning, attentional biases develop that are the result of implicit, unintentional, automatic learning.

The question is whether this type of learning is truly implicit and automatic or whether at least some awareness of the regularities present in the visual field plays a role in developing the biases. Several studies have investigated the question of whether conscious awareness is needed for extracting statistical regularities from sensory input across time. In triplet learning, some have shown that statistical learning is largely implicit. For example, participants who have shown a better performance on structured versus random stimuli were unable to indicate the underlying statistical patterns in the postexperiment familiarity test (Turk-Browne et al., 2005). Also, direct instruction to the underlying regularities had little effect. Participants who were informed about the statistical regularities contained in the stream performed no better than participants who were simply passively exposed to the underlying regularities (Arciuli et al., 2014; Song et al., 2007). On the other hand, it is feasible that information that is typically extracted through passive statistical learning can be acquired through more active learning strategies. For example, in a study by Batterink et al. (2015), in which observers were exposed to a continuous stream of repeating nonsense words, it was shown that a group that received explicit instructions responded faster than those that received no instructions. Moreover, the P300 potential of the EEG signal was larger to predictable words for the explicitly trained group than for the implicit group, which seem to suggest that the explicit group invested more effort. Also, there are studies showing that awareness does play a role in statistical learning. For example, it was reported that participants who showed more awareness of the underlying statistical patterns or were informed of the presence of the regularity showed the largest learning effects in terms of behavioral response times and ERPs (Rüsseler et al., 2003; Singh et al., 2017). Finally, it is also possible that explicit instructions result in worse performance. For example, in the domain of learning artificial grammar, relative to implicit instructions, participants that received clear and explicit instructions performed significantly worse that those participants that did not received explicit instructions (Reber, 1976).

Compared with sequential triplet or non-sense (artificial) word learning, in visual search, explicit knowledge regarding the regularities present in the display may even be more difficult to extract because it is well known that participants have little awareness of where attention (Wolfe, 1999) or their eyes (Clarke et al., 2017; Võ et al., 2016) have been. For example, Proulx (2011) found that 34 of 40 participants’ attention was captured by a salient item, even though none of the participants reported that the salient items was distracting. In a study measuring eye movements, Theeuwes et al. (1998) showed that observers were basically unaware that their eyes were captured by irrelevant salient onset distractors. Even though eye movement measurements clearly showed that the eyes of the observers went to the onset distractor, at the end of the experiment, when explicitly asked, all observers indicated that they had never looked at the onset distractor, suggesting that none of the observers were aware of their eyes being captured (see also Burra & Kerzel, 2014).

One way to make participants aware of where their attention has been is simply by asking in each trial to report where their attention has been. For example, in a recent eye-movement study, Adams and Gaspelin (2021) had participants perform an oculomotor capture task in which they had to find a target shape while ignoring a salient distractor. On each trial, participants had to report whether they thought their eyes were captured by the distractor or not. When the eyes were captured, participants were much more likely to report this than when the eyes were not captured. These findings indicate that by asking participants explicitly each trial, participants may become aware of where attention or the eyes have been. Also, participants become aware of shifts of attention towards a salient singleton when they are asked to indicate each trial whether they believed their attention was captured by the salient singleton or not (Adams & Gaspelin, 2020).

In the current study, we wanted to create conditions in which participants would become aware of the statistical regularities introduced, or not aware at all. We used the same task as Wang and Theeuwes (2018b), in which a salient distractor was presented much more often in one location than in all other locations. In these types of tasks, participants learn the regularities present in the display biasing attention such that the location that is most likely to contain a distractor becomes suppressed relative to all other locations. In addition to performing this task, for different groups of participants, we asked participants to indicate either the location of distractor or the location of the target on the preceding trial. Because the regularity concerns the distractor, we assumed that those participants that need to report on each trial the location of the distractor will become very much aware of the statistical regularity present in the display. However, those participants that need to report the location of the target singleton (which was random), will not be monitoring the regularity of the distractor, and therefore will be fully unaware of this regularity.

Given these conditions, we ask the question whether learning is affected by whether the regularities present in the display are implicit (unaware) or are explicit (aware). We determine the extent to which learning is successful on the basis of the suppression of the high-probability location. It is possible that relative to implicit learning, becoming explicitly aware of the regularities gives better performance (Batterink et al., 2015) or worse performance (Reber, 1976). Also, it is feasible that explicit knowledge regarding the regularities has basically no effect on performance.

## Experiment 1

In Experiment 1, after each search display, one group of participants had to report the location of the target and another group of participants had to report the location of the distractor. The report condition was a between-subjects variable.

### Method

#### Participants

For the learned suppression, G*Power software (Faul et al., 2007) indicated that a sample of 36 participants for each group would provide power of 0.9 to detect a significant medium-size (*f* = 0.25) within-subjects difference in response time. Besides, there was 0.95 chance of correctly rejecting the null hypothesis of no significant interaction between conditions with a total of 44 participants. Therefore, a planned number of 80 participants (*M*_age_ = 24.35 years, *SD* = 2.7 years, 40 females) were recruited online through the website Prolific (www.prolific.co). Five participants were replaced because of low accuracy on the search task (mean accuracy <65%). Forty participants were assigned to each group, and they were provided with 3.5£ for completing the whole experiment. All participants were right-handed, had normal or corrected-to-normal vision, and were naïve to the purpose of the experiment, they also provided informed consent before the experiment. Only participants who at least acquired an undergraduate degree and between the ages of 18 to 30 years were allowed to participate. The ethical committee of the Faculty of Behavioral and Movement Sciences of Vrije Universiteit Amsterdam approved the study.

#### Apparatus, stimuli, design, and procedure

The experiment was created in OpenSesame (Mathôt et al., 2012) using OSweb, and run using JATOS (Lange et al., 2015). Because the experiment took place online, some factors (e.g., lighting and seating conditions) could not be controlled.

**Fig. 1:**
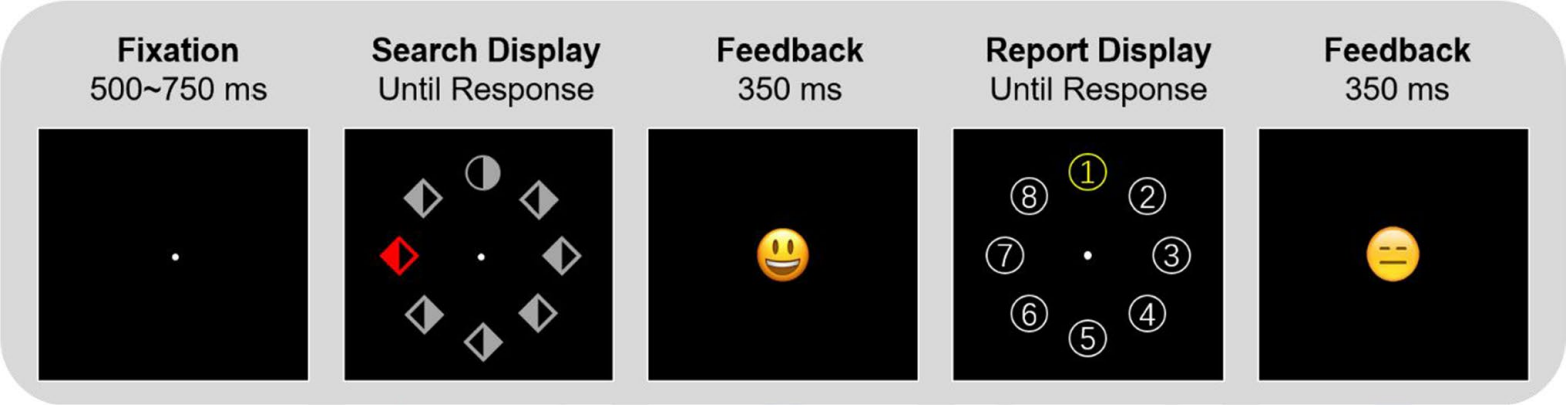
Experiment procedure of Experiment 1. Participants first needed to search for a unique shape target while ignoring the color singleton distractor. The search display was presented for 3 s or until response. The search display was followed by a report display. One group of participants were asked to report the location of target by pressing the corresponding number on the screen, while another group of participants needed to report the location of the color singleton distractor. Participants received an emoticon as feedback after each response. (Color figure online)

As shown in Fig. 1, each trial started with a 500–750-ms white fixation dot on a black background (radius 5 pixels), followed by a visual search display. This display contained eight shapes (seven diamonds and one circle, or vice versa; 130 pixels in diameter) with either the left or right half filled, all placed on an imaginary circle centered (radius 224 pixels) on fixation.

The target was the unique shape item (e.g., a circle among diamonds) and was always gray. Here, participants needed to respond to the unique shape by pressing the left or right key to indicate which half of the shape was filled within 3 s. Participants received a smiley or frowny face after the response as the search feedback.

Crucially, there were three separate search conditions. In no-distractor condition (30% of trials), all eight shapes were in gray (RGB: 128/128/128). In the high-probability distractor condition (35% of trials), seven of them were in gray while the remaining one was either green (0/128/0) or red (255/0/0), as the color singleton distractor. The high-probability location remained the same for any given participant. In the low-probability distractor condition (35% of trials), there was also one distractor, either red or green, among seven gray shapes, but this distractor could be located at any other seven locations except the high-probability one. Overall, the distractor was presented 7 times more likely at the high-probability location than at any of the low-probability locations.

Following a visual search trial, the report display, consisting of eight white-rimmed circles (radius 40 pixels), with 1–8 digits filled in, were placed at the exact same locations as the preceding search items. In the report target location group, participants needed to indicate the target location of the preceding search trial by pressing the corresponding number within 3 s. In the report distractor location group, participants were asked to indicate the location of the color singleton distractor of the preceding trial by pressing the corresponding number. If no distractor was present, participants responded “0.” They would also receive a smiley or frowny face as the feedback after the response. The intertrial interval was 500 ms.

Participants in this study first received 20 trials as practice, with distractor randomly presented at every location. The main experiment consisted of four blocks of 80 trials, lasted for around 30 min. Participants were encouraged to take a break between blocks and also received feedback on their mean RT and accuracy after each block. At the end of the experiment, participants of the two different groups were asked to answer three same questions. First, they were asked whether they noticed that there was a specific location at which distractor appeared more often (yes or no). Second, they needed to indicate the exact location by pressing the corresponding number (1–8; the display here was similar to the report display). For the last question, we asked them how confident they were about their answers (scaled from 1 to 5).

### Results

#### Awareness of the regularity

The mean accuracy of report distractor location after each search trial was 97.65%, *SD* = 1.6%; for report target location responses, the mean accuracy was 97.05%, *SD* = 2.2%.

As shown in Table 1, participants’ responses to the last three questions differed depending on which location they needed to report after the search trial. Participants in the report target group hardly noticed the regularity of distractor, only nine of 40 participants answered the correct high-probability distractor location with a relatively low confidence; while almost all participants (37/40) in the report distractor group could indicate the correct location and were quite confident of their answers. These results show that our manipulation of awareness of the regularity between groups was successful.

**Table 1:** Awareness assessment in Experiment. 1

	Report target location group	Report distractor location group
Total *N*	40	40
Report *yes*	7 (17.5%)	34 (85%)
Report *Correct* location	9 (22.5%)	37 (92.5%)
Likers (average)*	1.85	3.62

*1 = *not sure at all*; 2 = *somewhat sure*; 3 = *neutral*; 4 = *very sure*; 5 = *definitely sure*

#### Search performance

For RT analysis, 4.59% error trials or trials with an RT larger than 2.5 standard deviations from the average response time per condition per participant or less than 200 ms were excluded.

**Fig. 2:**
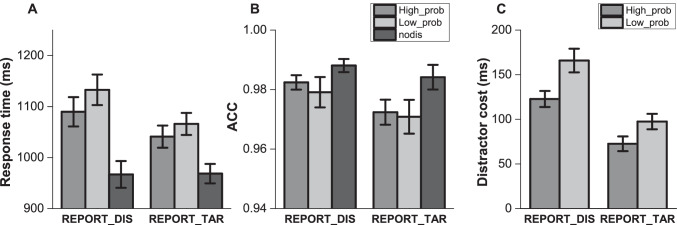
mean RTs (**a**), accuracy (**b**) and distractor cost (**c**) in Experiment 1. Standard error bars are shown

Mean RTs and accuracy of search task are presented in Fig. 2a and b. An analysis of variance (ANOVA) on mean RT with report condition (report target location vs. report distractor location) and distractor condition (high-probability locations, low-probability locations, and no distractor) as factors revealed a main effect of distractor condition, *F*(2, 156) = 188.78, *p* < .001, η_p_^2^ = .708, as well as a significant interaction, *F*(2, 156) = 12.672, *p* < .001, η_p_^2^ = .14, but not for the report condition, *F*(1, 78) = 1.232, *p* = .27, η_p_^2^ = .016.

Subsequent analysis showed that in both report conditions, participants were slower when a distractor was present than when it was absent: report target, *t*(39) = 6.417, *p* < .001; report distractor, *t*(39) = 5.626, *p* < .001, showing the classic attentional capture effect. Also, attentional capture was attenuated when the distractor was presented at the high-probability location relative to the low-probability location, report target, *t*(39) = 3.475, *p* < .001; report distractor, *t*(39) = 3.604, *p* < .001, replicating previous findings (Wang & Theeuwes, 2018b), suggesting suppression of the high-probability location.

The analysis of accuracy data only revealed a main effect of distractor condition, *F*(2, 156) = 9.082, *p* < .001, η_p_^2^ = .104, with higher accuracy in distractor-absent condition.

We assessed whether the SL effect differed depending on the manipulation of awareness of the distractor. Here, we calculated the distractor cost (mean RTs in the high-probability, low-probability location minus no-distractor condition) and ran a 2 (report condition) × 2 (distractor location) repeated-measures ANOVA (see Fig. 2c). There was a main effect of report condition, *F*(1, 78) = 23.246, *p* < .001, η_p_^2^ = .23, reflecting overall larger interference of distractor induced by the manipulation of distractor awareness. The main effect of distractor location was also significant, *F*(1, 78) = 23.798, *p* < .001, η_p_^2^ = .234, with smaller distractor cost when the distractor in the high-probability location relative to when the distractor in low-probability locations.

Crucially however, there was no significant interaction between these factors, *F*(1, 78) = 1.699, *p* = .196, η_p_^2^ = .021. The Bayesian statistics (BF01 = 2.04) using JASP (JASP Team, 2018) showed evidence that the data are better represented by the two main effects model than by the model that also includes the interaction. The absence of this interaction indicates that participants in both report groups equally effectively suppressed the location that was most likely to contain a distractor. It suggests that awareness of the regularity had no significant effect on learning to suppress the location that most likely contained a distractor.

To ensure that awareness did not play a role, we conducted an additional analysis in which we excluded the data from those nine participants of the report target group who indicated the correct high-probability distractor location and those three participants of the distractor report group who failed to indicate the correct high-probability distractor location. Again, the critical interaction failed to reach significance, *F*(1, 66) = .842, *p* = .362, η_p_^2^ = .013. The Bayesian statistics also supported the two main effects model 2.8 times better than the model that also included the interaction.

We compared the learning effect between aware and unaware groups regardless of task type. Based on Table 1, seven participants from the report target location group and 34 participants from the report distractor location group can be considered as the *aware group*, since they reported in the final questions that they were aware of the distractor location probability. The remaining 39 participants were assigned to the *unaware group*. The results again support the idea that awareness did not play a role in SL. We found significant main effects for distractor condition, *F*(1, 78) = 23.235, *p* < .001, η_p_^2^ = .23, and group, *F*(1, 78) = 8.694, *p* = .004, η_p_^2^ = .10, but not for an interaction, *F*(1, 78) = 0.062, *p* = .804. The Bayesian statistics (BF01 = 4.162) shows evidence that the data is better represented by two main effects model than the model that also includes the interaction.

#### Learned suppression effect across blocks

**Fig. 3:**
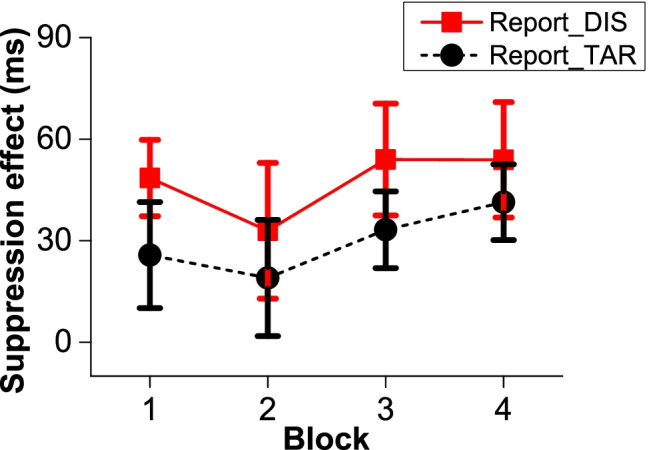
The mean learned suppression under the report distractor location condition and the report target location condition across blocks. Standard error bars are shown

As shown in Fig. 3, we ran a 4 (block) × 2 (report condition) ANOVA on the suppression effect (mean RTs in the low-probability condition minus high-probability condition) and did not find a significant main effect nor an interaction between these factors (*F*s < 1.535, *p*s > .219). The learned suppression showed no difference between the two groups. It is clear that learning happened relatively fast and was stable across the experiment.

#### Repeated suppression effect

To verify whether the suppression effect was not due to the trial-to-trial distractor location repetition, we reran the analysis above after excluding all trials in which the distractor was presented at the same location as in the immediately preceding trial and all results were replicated. An ANOVA on distractor cost showed a main effect of report condition, *F*(1, 78) = 19.312, *p* < .001, η_p_^2^ = .198, distractor location, *F*(1, 78) = 12.171, *p* = .001, η_p_^2^ = .135, but, again, no interaction, *F*(1, 78) = 1.342, *p* = .25, η_p_^2^ = .017.

### Discussion

The results of Experiment 1 are clear. Regardless of whether participants were aware or unaware of the regularity, learning to suppress the high-probability location was equally effective. It seems that, at least for this type of task, for learning to occur, it does not matter whether participants explicitly know the regularity or whether they have no explicit knowledge about it.

Even though the absence of an interaction indicated that learning to suppress was not affected by our report manipulation, it is clear that our dual task instruction had a main effect on RT (Fig. 2c). Indeed, in the distractor-present condition, participants were reliably slower when they had to report the location of the distractor relative to when they had to report the location of the target. To ensure that the current findings are not due to this dual task requirement to report the distractor or target after each search trial, in Experiment 2 we had participants report either the target or the distractor only every 40 trials. As the total number of trials was 320, this implies that during 97.5% of trials there was no dual task requirement.

## Experiment 2

Experiment 2 was identical to Experiment 1, except that participants reported either the most likely target or distractor location every 40 search trials instead of on each trial.

### Method

#### Participants

A new set of 80 participants (*M*_age_ = 22 years, *SD* = 2.52 years, 32 females) were recruited online through the website Prolific (www.prolific.co). One participant was replaced because of low accuracy on the search task (mean accuracy <65%). Forty participants were assigned to each group, and they were provided with 2.7£ for completing the whole experiment. All participants were right-handed, had normal or corrected-to-normal vision, and were naïve to the purpose of the experiment, they also provided informed consent before the experiment.

#### Materials and methods

The methods and analyses were the same as in Experiment 1, except for the following changes. Instead of indicating the distractor or target location after each search trial, here, we asked participants to indicate a location that contained the distractor or target most often during the last 40 trials (during instruction, participants were informed that they would be asked after 40 trials). The main experiment consisted of 320 trials, and lasted for about 20 min. Participants first received 20 trials as practice, with the distractor randomly presented at every location. For every 40 trials, participants in two different groups were instructed to indicate the location related to the distractor or target distribution, then take a break and receive feedback on their mean RT and accuracy. At the end of the experiment, participants also needed to answer the same three final questions as in Experiment 1.

### Results

#### Awareness of the regularity

As shown in Table 2, participants’ responses to the last three questions differs depending on the manipulation of report distractor or target regularity. Participants in the report distractor regularity group were more aware (75%) of the high-probability distractor location and relatively more confident about their answers than those in the target report group (30%). During the experiment, each participant was required to indicate the most likely target or distractor location during the previous 40 trials. In the target report group, there was not a “most likely target location,” and therefore it is not surprising that performance was at change level (average 1.1 times of 8). For the group reporting the most likely distractor location consistent with this distractor regularity, the average times of answering the high-probability distractor location was 3.85, which was significantly above chance level.

**Table 2:** Awareness assessment in Experiment 2

	Report target location group	Report distractor location group
Total *N*	40	40
Report *yes*	12 (30%)	28 (70%)
Report *Correct* location	12 (30%)	30 (75%)
Likers (average)*	1.9	3.02
Report during correct location during experiment	1.1/8	3.85/8

*1 = *not sure at all*; 2 = *somewhat sure*; 3 = *neutral*; 4 = *very sure*; 5 = *definitely sure*

#### Search performance

For RT analysis, 10.73% error trials or trials with an RT larger than 2.5 standard deviations from the average response time per condition per participant or less than 200 ms were excluded.

**Fig. 4:**
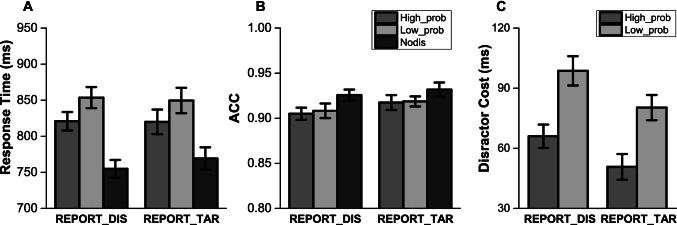
mean RTs (**a**), accuracy (**b**) and distractor cost (**c**) in Experiment 2. Standard error bars are shown

As shown in Fig. 4a, a 2 (report condition) × 3 (distractor condition) repeated-measures ANOVA on RTs revealed a main effect of distractor condition, *F*(2, 156) = 209.9, *p* < .001, η_p_^2^ = .729. There was no difference between two different report condition groups, *F*(1, 78) = .023, *p* = .879, nor a reliable interaction effect, *F*(2, 156) = 2.455, *p* = .091, η_p_^2^ = .031.

Subsequent analyses again showed that in both report conditions, participants were slower when a distractor was present than when it was absent: report target, *t*(39) = 11.488, *p* < .001; report distractor, *t*(39) = 14.017, *p* < .001—the classic attentional capture effect. Also, attentional capture was attenuated when the distractor was presented at the high-probability location relative to the low-probability location, report target, *t*(39) = 5.293, *p* < .001; report distractor, *t*(39) = 5.392, *p* < .001, showing the SL of the high-probability distractor location.

The same analyses conducted on accuracy data only revealed a main effect of distractor condition, *F*(2, 156) = 7.55, *p* = .001, η_p_^2^ = .088, with higher accuracy when the distractor was absent (see Fig. 4b).

We conducted a 2 (report condition) × 2 (distractor location) repeated-measures ANOVA on distractor cost (see Fig. 4c) to better assess the SL effect of distractor location for the different awareness groups. The results were similar as in Experiment 1, revealing a main effect of distractor location, *F*(1, 78) = 57.041, *p* < .001, η_p_^2^ = .422, with smaller distractor cost when the distractor in the high-probability location relative to when the distractor in the low-probability locations. There was also a main effect of the report condition, *F*(1, 78) = 4.214, *p* = .043, η_p_^2^ = .051, as for the first experiment, the presence of distractor in the report distractor regularity group caused relatively larger interference than in the report target regularity group. Note that the main effect of the report condition is mainly due to the difference on the no-distractor condition. As shown in Fig. 4a, compared with the report target regularity group, participants in the report distractor group were relatively faster when the distractor was absent. One very plausible explanation is that they do not need to keep a distractor location in mind while the other group still needs to memorize a target location.

Critically, again the interaction was not significant, *F*(1,78) = .137, *p* = .712. The Bayesian statistics also supported the two main effects model 3.7 times better than the model that also includes the interaction. The results show that regardless of the level of awareness of the regularity present in the display, learning to suppress the high-probability location is equally effective.

Again, after excluding trials in which the location repeated, we obtained the same results (two main effects: *F*s > 6.517, *p*s < .013), interaction, *F*(1, 78) = .918, *p* = .341, suggesting that these effects are not mainly driven by intertrial priming.

### Discussion

Experiment 2 basically replicates Experiment 1: Regardless of whether participants are aware of the regularity present in the display, learning is equally effective. While in Experiment 1, participants were required to report a location after each trial, in Experiment 2 they only reported it every 40 trials. This ensures that the effect reported in Experiment 1 was not due to the dual task requirement of reporting the location of the target or distractor on each trial.

The postexperiment questionnaire and the probe questions after every 40 trials revealed that participants in the report distractor group showed more awareness of distractor regularity than the participants in report target group.

## General discussion

The present findings are clear. In two experiments, we showed that the extent to which participants are aware of the regularities present in the display had no effect on learning to suppress the location that was most likely to contain a singleton distractor. It suggests that explicit knowledge that one location is more likely to contain a distractor does not affect the extent to which this location is suppressed.

These current findings are consistent with Wang and Theeuwes (2018b), who did not explicitly manipulate awareness, but on the basis of a postexperiment questionnaire split up the data into a group of participants that showed some awareness and a group that showed little awareness. There was no difference in the amount of suppression between these groups, suggesting that awareness had little, if any, effect on learning. Note, however, that in Wang and Theeuwes (2018b) the difference in awareness between the two groups was minimal, which make it difficult to draw firm conclusions on that post hoc data alone. However, the current result provides convincing evidence that explicit knowledge regarding the regularities in the display does not affect learning.

Our findings also show that the requirement to report the location of the distractor results in overall more attentional capture than when one has to report the location of the target. Even though this did not affect learning, relative to the no-distractor condition, participants were slower in the report distractor than in the report target condition. It is not immediately clear why this effect is found, but it is likely that attention dwells longer at the distractor location when one is required to monitor and report back this location. If attention dwells longer at the distractor location before it is redirected to the target location, the effect of attentional capture gets larger (Born et al., 2011). Another possibility is that reporting the location of a task-irrelevant distractor after each trial requires more attentional resources than reporting the location of the task-relevant target.

Our manipulation of awareness by instructing different groups to either monitor the target or monitor the distractor turned out to be very effective. Previous studies (Arciuli et al., 2014; Rüsseler et al., 2003; Song et al., 2007) have used a different approach and informed participants beforehand about the statistical regularity present in the display. For example, Song et al. (2007) used a modified version of the alternating serial response time task (ASRT) to dissociate automatic and intentional learning of triples. Before the experiment, they directly told one group of participants that one type of target (i.e., gray), always followed a pattern, whereas another group of participants were not informed of this regularity. The results showed that implicit learning occurred to the same extent—people generally responded faster to the high-frequency triples compared with the low-frequency triples, whether or not they had prior explicit knowledge of this pattern. We considered this explicit instruction approach as less optimal as in our experiment the regularity concerned the distractor and not the target. Explicit inhibition can lead to opposite effects in which observers attend those items that they are asked and trying to ignore (“white bear” effect; Tsal & Makovski, 2006). Also, a previous study in which participants received explicit instructions to actively suppress the distractor location showed that explicit instructions are not effective. This previous study showed that on the basis of instruction alone, participants were not able to suppress the location of the distractor (Wang & Theeuwes, 2018a). Also, we consider one group of participants having explicit instructions and one group without instructions as less optimal as the mere instruction alone could be the reason for finding differences between these groups.

Even though some have argued that there is no clear distinction between implicit and explicit learning (Destrebecqz & Cleeremans, 2003), it is evident that in our study, in the report distractor condition, the distractor becomes task relevant, and because of this, attention will be directed to the distractor location in top-down way. Indeed, as outlined, the overall larger attention capture in the report distractor condition is probably due to some consolidation process after attention has been captured. Even though in this condition following attentional capture, there is top-down consolidation and storage of the distractor location, our results show that this top-down process does not result in stronger suppression compared with the report target location condition. This finding is consistent with a recent study that showed that visual statistical learning can occur in the absence of top-down attention (Duncan & Theeuwes, 2020). Indeed, the Duncan and Theeuwes (2020) study shows that participants learn particular regularities simply because they are exposed to them under conditions in which attending these regularities is unrelated to the current task and goals. Similarly, in another study, it was shown that participants learn to suppress a distractor location even when working memory is fully loaded, suggesting that extracting regularities from the environment does not rely on active or explicit control resources (Gao & Theeuwes, 2020).

Even though it is clear that in the report distractor condition, directing attention to the distractor location is task relevant, it should be realized that in the report target condition, attention is also captured by the distractor in an exogenous way (Theeuwes, 1991, 1992). Our data suggest that the mere capture to the location of the distractor is sufficient for learning to occur, as the suppression in this condition was just as strong as in the condition in which capture by the distractor was task relevant. We assume that capture of attention to the location to the distractor is needed for learning to occur (see also Failing & Theeuwes, 2017, for a similar argument).

The current study suggests that explicit knowledge regarding the distractor and awareness of the regularity does not result in stronger suppression than the mere passive statistical learning without explicit knowledge and awareness. Even though this is fitting with the idea that statistical learning is automatic and relies on implicit associations, there is some evidence from experiments investigating skill acquisition that in a serial reaction time task, explicit knowledge of the repetitions may boost performance, as long as participants are also able to acquire implicit knowledge in parallel (Willingham et al., 1989).

In sum, the current study shows that explicit knowledge and awareness regarding the regularities present in the display does not affect learning to suppress a distractor. It is consistent with the notion that people are able to extract implicit representations automatically, without conscious effort or awareness.
